# Comparison of Dual Monoclonal Antibody Therapies for COVID-19 Evolution: A Multicentric Retrospective Study †

**DOI:** 10.3390/v16101542

**Published:** 2024-09-29

**Authors:** Karen Zafilaza, Jonathan Bellet, Aurélie Truffot, Vincent Foulongne, Manuela Mireille Onambele, Maud Salmona, Camille Vellas, Claire Périllaud-Dubois, Audrey Mirand, Elisabeth André-Garnier, Enagnon Kazali Alidjinou, Ségolène Brichler, Honorine Fenaux, Magali Bouvier-Alias, Cédric Hartard, Céline Dorival, Fabrice Carrat, Anne-Geneviève Marcelin, Karl Stefic, Cathia Soulie

**Affiliations:** 1 Laboratoire de Virologie, Hôpitaux Universitaires Pitié-Salpêtrière–Charles Foix, Institut Pierre Louis d’Epidémiologie et de Santé Publique, Institut National de la Santé et de la Recherche Médicale, Sorbonne Université, Assitance Publique-Hôpitaux de Paris, 75013 Paris, France; anne-genevieve.marcelin@aphp.fr (A.-G.M.); cathia.soulie-ext@aphp.fr (C.S.); 2 Département de Santé Publique, Hôpital Saint-Antoine, Institut Pierre Louis d’Epidémiologie et de Santé Publique, Institut Nationale de la Santé et de la Recherche Médicale, Sorbonne Université, Assistance Publique-Hôpitaux de Paris, 75012 Paris, France; jonathan.bellet@iplesp.upmc.fr (J.B.); celine.dorival@iplesp.upmc.fr (C.D.); fabrice.carrat@iplesp.upmc.fr (F.C.); 3 Centre National de la Recherche Scientifique, Commisariat à l’Energie Atomique, Institut de Recherche Interdisciplinaire de Grenoble Institut de Biologie Structurale, University Grenoble Alpes, 38000 Grenoble, France; atruffot@chu-grenoble.fr; 4 Laboratoire de Virologie, Centre Hospitalier Universitaire Montpellier, 34090 Montpellier, France; v-foulongne@chu-montpellier.fr; 5 Laboratoire de Virologie, Unité Mixte de Recherche 1137 Infection Antimicrobials Modelling Evolution, Institut Nationale de la Santé et de la Recherche Médicale, Université Paris Cité, Assistance Publique -Hôpitaux de Paris Nord Hôpital Bichat–Claude-Bernard, 75018 Paris, France; manuellamireille.onambeleguinidi@aphp.fr; 6 Laboratoire de Virologie, Unité 941, Institut Nationale de la Santé et de la Recherche Médicale, Hôpital Saint-Louis, Université de Paris, Assistance Publique-Hôpitaux de Paris, 75010 Paris, France; maud.salmona@aphp.fr; 7 Laboratoire de virologie, Centre Hospitalier Universitaire Toulouse Purpan, Institut Nationale de la Santé et de la Recherche Médicale Unité Mixte de Recherche 1291, 31300 Toulouse, France; camille.vellas@inserm.fr; 8 Laboratoire de Virologie, Hôpital Universitaire Saint-Antoine, Institut Pierre Louis d’Epidémiologie et de Santé Publique, Institut Nationale de la Santé et de la Recherche Médicale, Sorbonne Université, Assistance Publique-Hôpitaux de Paris, 75012 Paris, France; claire.dubois@aphp.fr; 9 Laboratoire de Virologie, Centre Hospitalier Universitaire de Clermont-Ferrand, 63003 Clermont-Ferrand, France; amirand@chu-clermontferrand.fr; 10 Laboratoire de Virologie, Centre Hospitalier Universitaire Nantes, 44000 Nantes, France; elisabeth.andre@chu-nantes.fr; 11 Laboratoire de Virologie, Centre Hospitalier Universitaire Lille, Université de Lille, 59000 Lille, France; enagnonkazali.alidjinou@chu-lille.fr; 12 Laboratoire de Virologie, Hôpital Avicennes, Assistance Publique-Hôpitaux de Paris, 93000 Bobigny, France; segolene.brichler@aphp.fr; 13 Laboratoire de Virologie, Centre Hospitalier Universitaire Paul Brousse, Assistance Publique-Hôpitaux de Paris, 94800 Villejuif, France; honorine.fenaux@aphp.fr; 14 Laboratoire de Virologie, Centre Hospitalier Universitaire Henri Mondor, Assistance Publique-Hôpitaux de Paris, 94000 Créteil, France; magali.bouvier@aphp.fr; 15 Laboratoire de Virologie, Centre Hospitalier Régional et Universitaire de Nancy Brabois, Laboratoire de Chimie Physique et Microbiologie pour les matériaux et l’Environnement, Centre National de la Recherche Scientifique, Université de Lorraine, 54500 Vandœuvre-lès-Nancy, France; c.hartard@chru-nancy.fr; 16 Laboratoire de Virologie, Centre Hospitalier et Régional Universitaire de Tours, Unité 1259, Institut Nationale de la Santé et de la Recherche Médicale, 37000 Tours, France; karl.stefic@univ-tours.fr

**Keywords:** COVID-19, SARS-CoV-2, monoclonal antibody therapy, spike gene, immune escape mutation

## Abstract

Background: Neutralizing antibodies targeting the SARS-CoV-2 Spike protein reduce COVID-19-related risk of hospitalization, particularly in high-risk individuals. The COCOPREV-R study aimed to evaluate and compare clinical outcomes in high-risk SARS-CoV-2 patients treated with dual monoclonal antibody therapies and to identify associated virological factors. Methods: The COCOPREV-R study retrospectively collected real-world data from high-risk patients receiving Bamlanivimab/Etesevimab or Casirivimab/Imdevimab dual monoclonal antibody therapies (22 February 2021 to 15 June 2021). Results: The study included 1004 patients with COVID-19, of whom 691 received Bamlanivimab/Etesevimab and 313 received Casirivimab/Imdevimab. The alpha variant represented 90.1% of those for whom data were available. The risk of hospitalization within 30 days was lower with Bamlanivimab/Etesevimab (12.7%, CI 95% [9.9–16.3%]) compared to Casirivimab/Imdevimab (28.4%, CI 95% [22.7–35.1%) (*p* < 0.001). The 30-day mortality rates were comparable between both groups (*p* = 0.982). Analysis of SARS-CoV-2 PCR negativity showed no difference between the two treatment groups (95.2% [93.0–96.9%] and 93.5% [89.1–96.6%] until day 30, *p* = 0.851 for Bamlanivimab/Etesevimab and Casirivimab/Imdevimab, respectively). Among persistently positive samples with available sequencing results (*n* = 43), Spike protein changes occurred only in Bamlanivimab/Etesevimab (42.9%) vs. Casirivimab/Imdevimab (0.0%) groups. Q493R (25.0%) and E484K (12.5%) were the most common mutations selected by Bamlanivimab/Etesevimab in follow-up samples. Other factors (immunodepression, comorbidities, and age) did not appear to be associated with the occurrence of Spike protein mutations. Conclusions: A higher rate of hospitalization was seen with Casirivimab/Imdevimab (RONAPREVE^®^) in comparison with Bamlanivimab/Etesevimab treatment, but with the emergence of Spike mutations only in the Bamlanivimab/Etesevimab group.

## 1. Introduction

Neutralizing antibodies targeting the SARS-CoV-2 Spike protein have been shown to reduce the risk of COVID-19-related hospitalization in patients at high risk of progression, such as elderly or immunocompromised patients [1].

Bamlanivimab/Etesevimab was developed by Eli-Lilly from convalescent COVID-19 patients and targets the SARS-CoV-2 Spike glycoprotein (S), which mediates viral entry into the host cell. In high-risk ambulatory patients, Bamlanivimab/Etesevimab led to a lower incidence of COVID-19-related hospitalization and death than did placebo, and accelerated the decline in the SARS-CoV-2 viral load [2,3]. 

In addition, Casirivimab/Imdevimab (RONAPREVE^®^) is a non-competitive monoclonal antibody combination that binds to two sites on the receptor-binding domain (RBD) of the SARS-CoV-2 glycoprotein, blocking viral entry into the host cell [4]. These potent neutralizing antibodies were developed by Regeneron Pharmaceutical. Casirivimab/Imdevimab reduced the risk of COVID-19-related hospitalization or death from COVID-19 or other causes, and this dual therapy resolved symptoms and reduced the SARS-CoV-2 viral load faster than placebo in a phase 3 study [5]. 

The COCOPREV-R (Prevention of complications of COVID-19 in high-risk individuals infected with SARS-CoV-2 who have received treatment under a Temporary Authorization for Use) was a retrospective study collecting data from patients receiving a dual monoclonal antibody therapy, Bamlanivimab/Etesevimab or Casirivimab/Imdevimab (RONAPREVE^®^) in real-world settings. At the time of the study, patients at high risk of severe disease progression were treated with Bamlanivimab/Etesevimab or Casirivimab/Imdevimab in accordance with French recommendations. These dual monoclonal antibodies were authorized for emergency use and administered to patients based on criteria. 

The main objective was to evaluate and compare the clinical outcomes (hospitalization and death rates) of SARS-CoV-2-infected patients at high risk of complications who were treated with two dual monoclonal antibody therapies. Virological factors, such as SARS-CoV-2 viral load, and emergence of resistance were also assessed. Viral load measurement is critical as prolonged viral replication increases the risk of mutation emergence.

## 2. Patients and Methods

### 2.1. Study Design

This retrospective COCOPREV-R study enrolled patients at a high risk of progression to severe COVID-19, with PCR-proven, mild-to-moderate disease not requiring hospitalization, in the first five days of symptoms, who were treated with dual monoclonal antibody therapy (Bamlanivimab/Etesevimab and Casirivimab/imdevimab (RONAPREVE^®^)) between 22 February 2021 and 15 June 2021. These treatments were given under a Temporary Authorization for Use and were administered under specific conditions to patients considered at risk due to immunosuppression and comorbidities.

In accordance with the current laws and regulations in France, the study was approved by CESREES (Comité éthique et Scientifique pour les recherches, les études et les évaluations dans le domaine de la santé) on 8 July 2021 and by CNIL (Commission nationale de l’Informatique et des Libertés) under number n°921282 on 16 July 2021. 

The Temporary Authorization for Use protocol defined, globally, which patients could receive these treatments, prioritizing those considered to be at high risk due to factors such as immunosuppression and other comorbidities. Inclusion of these patients depended set criteria, which were as follows: (1) patients with compromised immunity due to a pathology or treatment (ongoing chemotherapy, solid organ transplant, allogeneic hematopoietic stem cell transplant, renal disease with eGFR < 30 mL/min or undergoing dialysis, systemic lupus or vasculitis with immunosuppressive treatment, corticosteroid treatment > 10 mg/day of equivalent prednisone for more than 2 weeks, immunosuppressive treatment (including corticosteroids, anti-CD20 treatment such as rituximab and treatment for autoimmune diseases)); (2) patients at risk of complications regardless of age (idiopathic pulmonary fibrosis, amyotrophic lateral sclerosis, rare liver diseases, including autoimmune hepatitis, myopathies with forced vital capacity < 70%, other rare diseases defined by Rare Disease Health Networks, trisomy 21); (3) patients between 70 and 80 years of age with at least one of the following diseases: obesity (BMI > 30), COPD and chronic respiratory failure, complicated hypertension, heart failure, diabetes type 1 and 2, chronic renal failure). When necessary, the choice between dual monoclonal antibody therapies was at the discretion of the physician.

Patients who did not meet these criteria were therefore not included. Contraindications to treatment were excessive treatment delay, duration of symptoms > 5 days, intensity of symptoms, severe form of COVID-19, detection of a variant carrying the E484K mutation, allergy to at least one of the treatment components, comorbidity that compromised the one-month prognosis or required surgical intervention within 7 days. 

### 2.2. Viral Load, Variant Identification, and SARS-CoV-2 Spike Gene Sequencing

Samples were collected on day 0 and then each time the patient attended a consultation.

Viral load was measured locally at each center by quantitative RT-PCR [6]. For the results’ analyses, the N gene was targeted. Each patient’s viral load was measured at the same center. SARS-CoV-2 variant determination and sequencing were performed until SARS-CoV-2 viral load was undetectable. SARS-CoV-2 variants were determined using RT-PCR screening techniques to detect mutations or combinations of mutations specific to each variant. Next-generation sequencing (NGS) using Gridion technology [7] and Sanger sequencing were also performed in each center on D0 and for the follow-ups. For the NGS, only the consensus sequence of the S gene was retained for the analysis of mutation emergence. The S gene mutation was considered new if not present in the sequence at baseline (D0). 

### 2.3. Data Management

Three data sources were used in this study. The first and second parts of the data were obtained from Roche and Eli-Lilly as part of the extended monitoring (hospitalization data, risk factor data, and treatment administration data) pending regulatory approvals. The two companies provided the name of the hospital, age, sex, risk factors for developing a severe form of COVID-19, date of treatment, and several elements collected at each visit: clinical signs, disease progression, hospitalization, adverse events, death, SARS-CoV-2 viral load, and variant identification. The third data source was sixteen virology laboratories of the ANRS-MIE network participating in an ANRS-MIE cohort. They filled an eCRF database with the hospital name, the age or the month and year of birth, sex, SARS-CoV-2 viral load, and S protein resistance mutations on D0 and at follow-up. 

The pharmaceutical and COCOPREV-R databases were matched for sex, date of birth (+/−1 year), and date of treatment administration (+/−5 days) using the SAS macro-program, GMATCH Program. 

The primary outcome was the proportion of patients with COVID-19-related hospitalization (if the patient was ambulatory) or prolonged hospitalization (>14 days if the patient was hospitalized) within one month of treatment administration. Secondary outcomes were the proportion of patients who died from COVID-19 and from other causes within one month of the onset of symptoms; the viral load dynamics and its determinants; time to negative conversion of nasopharyngeal SARS-CoV-2 PCR; and characterization of resistant variants and the determinants associated with their emergence. 

### 2.4. Data Analysis

Baseline characteristics were described by the median and interquartile range for quantitative variables, and by frequency and percentage for categorical variables. Comparison of baseline characteristics between the two treatments and according to whether a new mutation occurred during follow-up was performed using the Mann–Whitney Wilcoxon test for quantitative data, and the chi-square test or Fisher’s exact test for categorical data. The alpha risk for the comparison tests was 5%, and the confidence intervals were estimated at the 95% confidence level. 

The Kaplan–Meier (KM) method was used to calculate the probability of hospitalization and the probability of death during follow-up, and comparisons were made using the log-rank tests. The information on hospitalization and death probability was recorded in the database with the date of occurrence during follow-up. In the case of hospitalization, the date of first hospitalization was used. In the absence of hospitalization, censoring was applied at the date of death, at the last known date of follow-up (before 30 days of follow-up), or after 30 days of follow-up. If there was no death, censoring was applied after 30 days of follow-up or at the last known date of follow-up (before 30 days of follow-up).

The two dual monoclonal treatments were compared to determine if one had a better effect and improved the risk of hospitalization and death during follow-up. A Cox model was used to assess and compare this risk (hazard ratio). A multivariate analysis was performed adjusting for baseline factors that showed a significant difference (*p* < 0.05) between the two groups at baseline (baseline characteristic). The aim was to compare the risk of hospitalization or death by considering the effect of these factors on the risk of hospitalization or death during follow-up. An additional factor was considered in the adjustment, the time between the onset of symptoms and the start of treatment, to ensure that this data did not influence the risk of hospitalization. 

Interval censoring methods were used to effectively deal with the time gap between the last positive PCR value and the first negative PCR value. For hospitalization and death data, information was censored from the date of the last update if no hospitalization or death was recorded. It is important to note that the factors examined in the Cox model had a complete dataset, with no missing values.

These analyses were performed using SAS version 9.4 software.

## 3. Results

Baseline characteristics of 1004 patients are shown in Table 1. Of these, 691 and 313 received Bamlanivimab/Etesevimab and Casirivimab/Imdevimab (RONAPREVE^®^), respectively, 31.1% (*n* = 312) of them were older than 80 years, and 54.3% (*n* = 545) had an immunodeficiency related to a pathology or a treatment. Of the 365 known SARS-CoV-2 variants, the Alpha variant was predominant (90.1%; *n* = 329), with 3.3% (*n* = 12) of the Wuhan, 1.9% (*n* = 7) of Beta, 0.6% (*n* = 2) of Gamma strains, and 4.1% (*n* = 15) of various other variants, variant 20A/440K and co-infections implying Alpha, Beta and Gamma variants, with no difference in distribution between the two treatment groups (*p* = 0.174).

**Figure 1:**
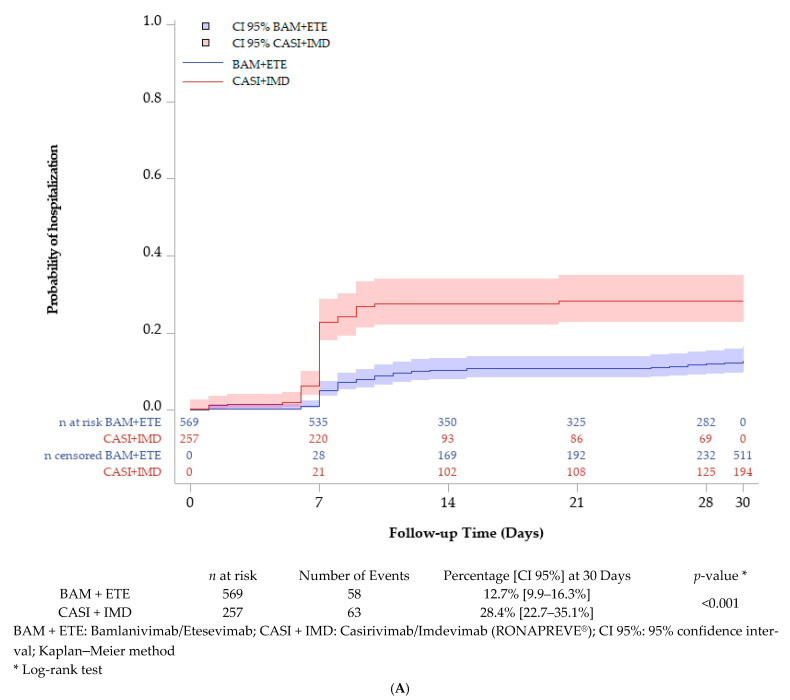

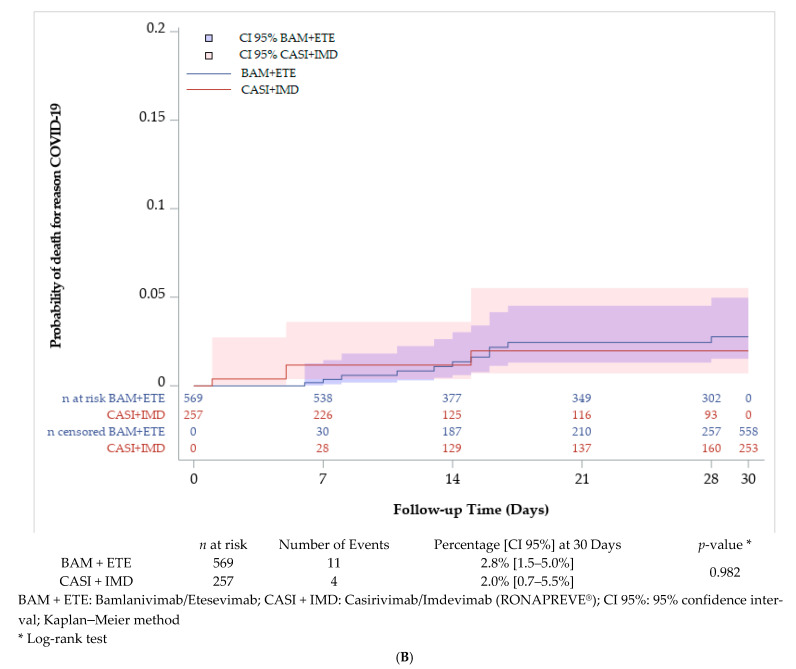

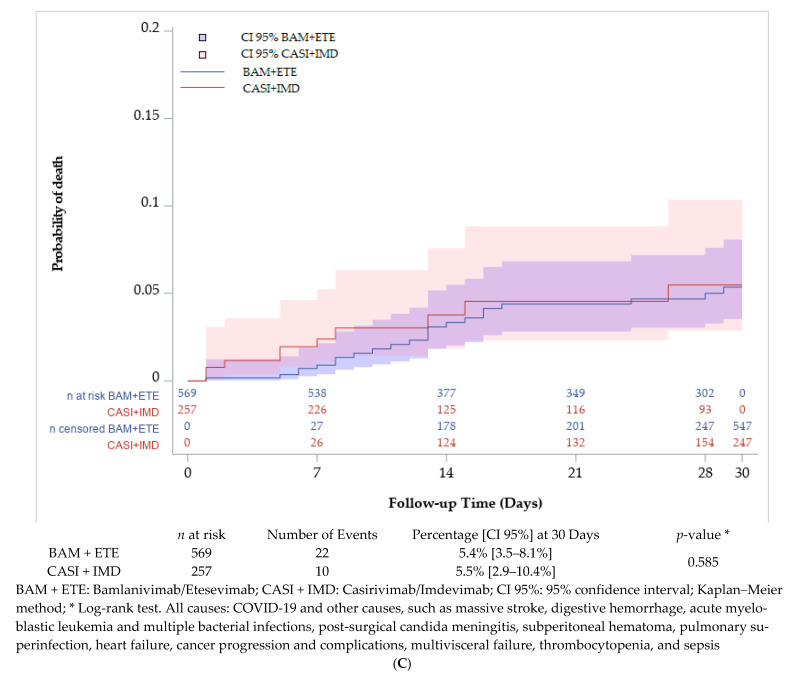
(**A**) Probability of hospitalization (COVID-19 cause) at 30 days of treatment administration (Kaplan–Meier method). Patients treated with Casirivimab/Imdevimab had a higher rate of hospitalization 30 days post-infusion (28.4% [22.7–35.1%]) compared to the Bamlanivimab/Imdevimab treatment group (12.7% [9.9–16.3%]). (**B**) Probability of death (COVID-19 cause) at 30 days of treatment administration (Kaplan–Meier method). The probability of death was low in both dual monoclonal antibody therapy arms. The proportion of deaths due to COVID-19 following infusion of Bamlanivimab/Etesevimab was 2.8% versus 2.0% in patients treated with Casirivimab/Imdevimab. (**C**) Probability of death (All causes) at 30 days of treatment administration (Kaplan–Meier method). The probability of death from all causes was similar between Bamlanivimab/Etesevimab and Casirivimab/Imdevimab (5.4% vs. 5.5%, *p* = 0.585)) until 30 days.

The probability of hospitalization (COVID-19 cause) at 30 days of treatment administration was significantly different between the Bamlanivimab/Etesevimab (12.7% [9.9–16.3%]) and Casirivimab/Imdevimab (RONAPREVE^®^) (28.4% [22.7–35.1%]) groups (*p* < 0.001) (Figure 1A). The effect of the treatment was confirmed in a multivariate analysis evidencing a HR of 3.0 [CI 95% 2.1–4.3] for Casirivimab/Imdevimab (RONAPREVE^®^) (*p* < 0.001), with no effect of immunodepression and comorbidities (*p* = 0.998 and 0.615, respectively) (Table 2). However, the probability of death from COVID-19 or all causes at 30 days post-treatment was similar between the two treatment groups (2.8% [1.5–5.0%] vs. 2.0% [0.7–5.5%], *p* = 0.982) COVID-19 cause; 5.4% [3.5–8.1%] vs. 5.5% [2.9–10.4%], *p* = 0.585, all causes for Bamlanivimab/Etesevimab and Casirivimab/Imdevimab (RONAPREVE^®^), respectively) (Figure 1B,C).

**Figure 2:**
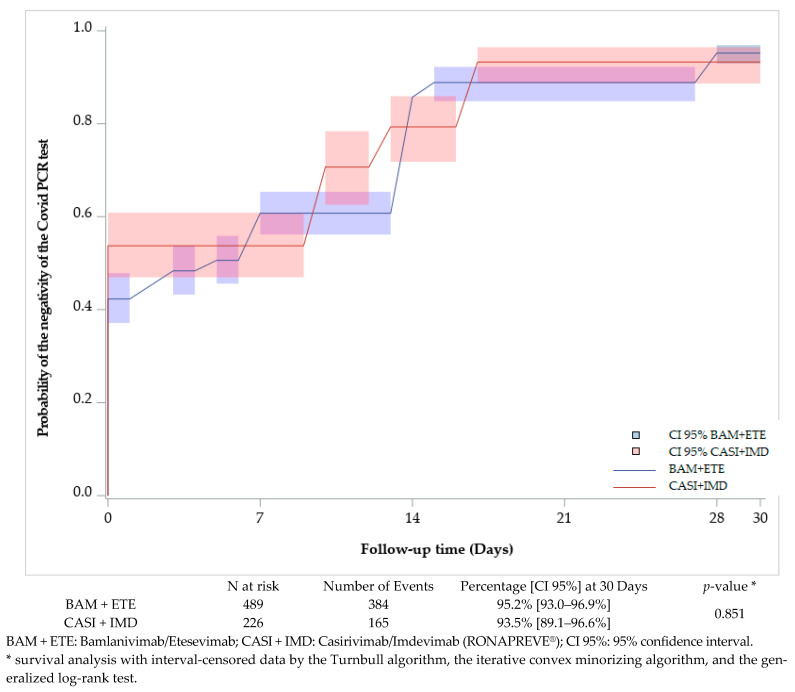
Analysis of the negativity of COVID-19 PCR tests (survival analysis method with interval-censored data) in the pharmaceutical company database. The proportions of negative PCR tests were similar between Bamlanivimab/Etesevimab and Casirivimab/Imdevimab (95.2% vs. 93.5% *p* = 0.851).

Analysis of SARS-CoV-2 PCR negativity showed no difference between the two treatments until day 30 (95.2% [93.0–96.9%] and 93.5% [89.1–96.6%], *p* = 0.851, for Bamlanivimab/Etesevimab and Casirivimab/Imdevimab (RONAPREVE^®^), respectively) (Figure 2).

Matching between the company’s databases and the virology database was successful in 198 patients (90 Bamlanivimab/Etesevimab and 108 Casirivimab/Imdevimab (RONAPREVE^®^)). S gene sequences were available for 102 patients on D0 (*n* = 56 Bamlanivimab/Etesevimab, *n* = 46 Casirivimab/Imdevimab (RONAPREVE^®^)). Due to negative SARS-CoV-2 PCR conversion, only 43 patients were followed up for S gene sequences (*n* = 28 Bamlanivimab/Etesevimab, *n* = 15 Casirivimab/Imdevimab (RONAPREVE^®^)) with a median follow-up of 7.5 (6.0–15.0) and 7.0 (3.0–10.0) days for Bamlanivimab/Etesevimab and Casirivimab/Imdevimab (RONAPREVE^®^), respectively. Despite the small number of patients with a follow-up, a higher number of mutations appeared in the Bamlanivimab/Etesevimab group (12/28, 42.9%) compared to the Casirivimab/Imdevimab group (RONAPREVE^®^) (0/15, 0.0%) group (*p* = 0.003) for the entire sequence of the Spike protein. The emerging mutations were A27S (*n* = 1), A67V (*n* = 1), S98F (*n* = 2), R102G (*n* = 1), A475V (*n* = 1), E484K (*n* = 2), Q493K (*n* = 1), and Q493R (*n* = 8).

None of the factors appeared to be associated with the occurrence of the Spike mutations (immunodepression, *p* = 0.160; comorbidities, *p* = 1.000; age, *p* = 0.401; and SARS-CoV-2 variant, *p* = 1.000).

## 4. Discussion

This retrospective, real-world study evaluated the efficacy of two approved dual SARS-CoV-2 antibody therapies in patients predominantly infected with the alpha variant. The analysis was performed retrospectively during a short period of time in the spring of 2021 between the approval of these two dual monoclonal antibody therapies and the start of the prospective ANRS 0003S COCOPREV study (September 2021), which explains the prevalence of the alpha variant.

A modest hospitalization rate was observed in patients treated with Bamlanivimab/Etesevimab and Casirivimab/Imdevimab (RONAPREVE^®^), with a lower hospitalization rate in the Bamlanivimab/Etesevimab group. This difference was not statistically explained in our study by the presence of more patients with pathological or treatment-related immunodeficiency or comorbidities in the Casirivimab/Imdevimab (RONAPREVE^®^) arm, although some differences in these two aspects were observed between the two arms at baseline, particularly in terms of being immunosuppressed or having received a solid organ transplant. However, similar rates of death from all causes or from COVID-19 were observed in the two treatment groups.

The emergence of mutations in the SARS-CoV-2 Spike sequences was only observed for the group of patients treated with Bamlanivimab/Etesevimab. The most common emergent mutations observed here were E484K and Q493R, both known to be the most important resistance mutations induced by this treatment [8,9,10,11]. The E484K and Q493R mutations occur in the Spike protein gene located on the surface of the virus. These changes affect the epitopes, making monoclonal antibodies less effective. The E484K mutation has previously been detected at the time of viral rebound, but not before treatment with monoclonal Bamlanivimab [12]. The Q493R mutation occurs after treatment with Bamlanivimab/Etesevimab [11,13].

E484K and especially Q493R are known to increase infectivity and immune escape. Indeed, in CRFK cells expressing ACE2, E484K mutants showed significantly reduced viral entry compared to the parental pseudotyped virus [14]. Another study showed a 3-fold increase in infectivity compared to the reference variant [15]. This observed difference in viral entry may be due to the different assay systems, including cell types, assay variance, or other factors. However, the E484K mutation is also associated with increased resistance to neutralizing antibodies. It alters the structure of the RBD, making antibody recognition more difficult [16]. Q493R increases the affinity of the RBD for the human ACE2 receptor, which can increase the infectivity of the virus. This mutation is also involved in the ability of the virus to escape certain immune responses, in particular, neutralizing antibodies produced by previous infections or vaccinations [11].

Bamlanivimab, which binds with high affinity to the receptor-binding domain of SARS-CoV-2, was derived from convalescent plasma of a patient with COVID-19 [17]. Both Bamlanivimab and Etesevimab target the receptor-binding domain of the Spike glycoprotein. They bind to different but adjacent epitopes within the RBD, blocking the interaction between the virus and the ACE2 receptor [18]. Casirivimab is an antibody whose epitope overlaps with the receptor-binding motif (RBM) within the RBD [19]. Imdevimab binds outside the RBM but still targets the RBD, making it effective against the Alpha and Delta variants [5,20]. The proximity of epitopes in the Bamlanivimab/Etesevimab combination may have contributed to the emergence of the E484K and Q493R mutations. In contrast, the greater distance between the epitopes of Casirivimab and Imdevimab may help prevent the development of resistance. 

A study of 13 patients, 8 of them treated with Casirivimab/Imdevimab, showed a single S RBD mutation, the E406G substitution [21]. Another study of 15 patients infected with the Alpha lineage showed no S RBD mutation in patients treated with Casirivimab/Imdevimab [22]. Resistance mutations to RONAPREVE treatment appear mainly in Delta and Omicron variant infections. Overall, Casirivimab/Imdevimab does not appear to induce mutations in the SARS-CoV-2 Alpha variant as we observed in our study, but rather in variants that appear later. Positions such as E406, G446, Y453, and L455 are known to increase immune escape and infectivity and are then found as lineage-defining mutations in subsequent variants, notably BQ.1 and XBB [1,23].

The emergence of new variants such as BQ.1.1, XBB and its sub-variants, and more recently, JN.1 and JN.1.1, have rendered monoclonal antibodies ineffective. Nevertheless, it is important to continue testing, in vitro, monoclonal antibodies against new variants. Bruel et al. showed that although Sotrovimab became ineffective against the Omicron BA.2 variant, it retained ADCC activity against BQ.1.1 and XBB.1.5 in immunocompromised patients [24]. The French health authority recommends PAXLOVID^®^ (Nirmatrelvir/Ritonavir) as a first-line treatment for non-oxygen-dependent patients and VEKLURY^®^ (Remdesivir) as a second-line treatment in hospital settings only. Patients treated with Paxlovid are severely immunocompromised, have underlying diseases or associated risk factors that can lead to severe COVID-19 disease, and are over 65 years old [25]. Overall, it is important to closely monitor potential resistance to COVID-19 treatment, as well as the evolution of variants, to constantly adapt the treatment.

There are several limitations to this study. First, the inherent nature of the retrospective study introduces several biases in both observation and interpretation. Indeed, the retrospective nature of the study is inherently associated with the risk of undetected confounders that could still account for the observed difference between the therapeutic regimens. Our work is limited by the lack of a control group, the relatively small number of sequences available on D0 and at follow-up, and furthermore, by the short duration of the follow-up (10 days). The results of this study are only valid for these two dual antibody therapies and this type of patient and cannot be extrapolated.

## 5. Conclusions

Despite some limitations in our study, both Bamlanivimab/Etesevimab and Casirivimab/Imdevimab (RONAPREVE^®^) treatments appear to be effective in reducing the rate of hospitalizations, with the Bamlanivimab/Etesevimab treatment showing greater efficacy. The emergence of resistance mutations was only observed in the Bamlanivimab/Etesevimab group. This suggests that the use of this type of treatment should be carefully monitored. As previously demonstrated, the use of monoclonal antibodies to treat patients at high risk of severe COVID-19 could promote the development of resistance to these treatments, and these resistance mutations have been found in several SARS-CoV-2 lineages, especially in several VOCs. So far, the circulating SARS-CoV-2 variants have not been sensitive to monoclonal antibodies, but the continued evolution of the variants may suggest the possible re-use of certain monoclonal antibodies.

## Figures and Tables

**Table 1:** Baseline characteristics of COVID-19-positive patients before monoclonal antibody treatment in the database of the pharmaceutical company.

Characteristics before Monoclonal Antibody Treatment	All *n* = 1004	Dual Therapy Bamlanivimab+ Etesevimab *n* = 691	Dual Therapy Casirivimab+ Imdevimab (RONAPREVE^®^) *n* = 313	*p*-Value
Age categories, *n* (%)				0.116
- Under 60 years old	287 (28.6)	184 (26.6)	103 (32.9)	
- between 60 and 80 years old	405 (40.3)	284 (41.1)	121 (38.7)	
- 80 years and more	312 (31.1)	223 (32.3)	89 (28.4)	
Sex, *n* (%)				0.748
- Female	524 (52.2)	363 (52.5)	161 (51.4)	
- Male	480 (47.8)	328 (47.5)	152 (48.6)	
Weight, kg, median (IQR)	73 (61–84)	73 (61–84)	71 (61–85)	0.341
Form of the COVID-19, *n* (%)				0.577
- Mild	853 (85.0)	590 (85.4)	263 (84.0)	
- Moderated	151 (15.0)	101 (14.6)	50 (16.0)	
Immune deficiency related to a pathology or treatments, *n* (%)				<0.001
- Yes	545 (54.3)	339 (49.1)	206 (65.8)	
- No	459 (45.7)	352 (50.9)	107 (34.2)	
Chemotherapy on-going, *n* (%)	185 (18.4)	119 (17.2)	66 (21.1)	0.143
Solid organ transplantation, *n* (%)	150 (14.9)	87 (12.6)	63 (20.1)	0.002
Immunosuppressive treatments *, *n* (%)	150 (14.9)	101 (14.6)	49 (15.7)	0.669
Corticosteroid treatment > 10 mg/day prednisone equivalent for more than 2 weeks, *n* (%)	67 (6.7)	47 (6.8)	20 (6.4)	0.809
Systemic lupus or vasculitis with immunosuppressive therapy, *n* (%)	20 (2.0)	13 (1.9)	7 (2.2)	0.709
Patients with comorbidities, *n* (%)				<0.001
- Yes	304 (30.3)	251 (36.3)	53 (16.9)	
- No	700 (69.7)	440 (63.7)	260 (83.1)	
Idiopathic pulmonary fibrosis, *n* (%)	6 (0.6)	3 (0.4)	3 (1.0)	0.383
Myopathy with a forced vital capacity, *n* (%)	3 (0.3)	2 (3.0)	1 (0.3)	1.000
Amyotrophic lateral sclerosis, *n* (%)	5 (0.5)	5 (0.7)	0 (0.0)	0.332
Trisomy 21, *n* (%)	3 (0.3)	3 (0.4)	0 (0.0)	0.556
Rare liver diseases including autoimmune hepatitis, *n* (%)	11 (1.1)	7 (1.0)	4 (1.3)	0.747
Other rare diseases defined by the FSMR, *n* (%)	23 (2.3)	14 (2.0)	9 (2.9)	0.405
Obesity, *n* (%)	102 (10.2)	85 (12.3)	17 (5.4)	<0.001
Diabetes, *n* (%)	120 (12.0)	102 (14.8)	18 (5.8)	<0.001
Heart failure, *n* (%)	65 (6.5)	57 (8.2)	8 (2.6)	<0.001
COPD and chronic respiratory failure, *n* (%)	44 (4.4)	39 (5.6)	5 (1.6)	0.004
Chronic renal failure	51 (5.1)	46 (6.7)	5 (1.6)	<0.001
Complicated high blood pressure, *n* (%)	68 (6.8)	59 (8.5)	9 (2.9)	<0.001
Time between symptoms and administration of treatment (days), median (IQR)	3 (2–4)	3 (2–4)	3 (2–4)	0.409
Time between symptoms and administration of treatment (days), *n* (%)				0.593
- 0	40 (4.0)	32 (4.6)	8 (2.6)	
- 1	142 (14.1)	102 (14.8)	40 (12.8)	
- 2	231 (23.0)	154 (22.3)	77 (24.6)	
- 3	271 (27.0)	185 (26.8)	86 (27.5)	
- 4	253 (25.2)	171 (24.8)	82 (26.2)	
- 5	67 (6.7)	47 (6.8)	20 (6.4)	
Reaction at the time of administration, *n* (%)				0.383
- No	998 (99.4)	688 (99.6)	310 (99.0)	
- Yes	6 (0.6)	3 (0.4)	3 (1.0)	
Infusion reaction/Hypersensitivity	3	1	2	
Others:	3	2	1	
- Agitation	1	1	0	
- Malaise-like reaction or chills	1	1	0	
- Vomiting at the end of the infusion	1	0	1	

IQR: interquartile; *p*-value: Mann–Whitney Wilcoxon test for quantitative data; chi-square test or Fisher exact test for categorical data. No missing value at baseline. * including corticosteroids, anti-CD20 treatment such as rituximab, and treatment for autoimmune diseases and inflammatory diseases.

**Table 2:** Comparison of hospitalization adjusted for risk factors and time between symptoms and treatment (days) (Cox Model).

		Univariate Analysis		Multivariate Analysis	
Characteristics	N	HR [CI 95%]	*p*-Value	HR [CI 95%]	*p*-Value
Treatment			<0.001		<0.001
- BAM + ETE	569	1		1	
- CASI + IMD	257	3.1 [2.1–4.4]		3.0 [2.1–4.3]	
Immune deficiency related to a pathology or treatment			0.178		0.955
- No	387	1		1	
- Yes	439	1.3 [0.9–1.8]		1.0 [0.7–1.5]	
Comorbidities			0.081		0.562
- No	587	1		1	
- Yes	239	0.7 [0.5–1.0]		0.9 [0.5–1.4]	
Time between symptoms and treatment (days)	826	1.11 [0.96–1.28]	0.159	1.1 [1.0–1.3]	0.151

BAM + ETE: Bamlanivimab/Etesevimab; CASI + IMD: Casivirimab/Imdevimab (RONAPREVE^®^); HR: Hazard Ratio; CI 95%: 95% confidence interval using Cox Model. Immune deficiency related to pathology or treatment of at least one pathology, including ongoing chemotherapy, solid organ transplantation, immunosuppressive treatments, including rituximab, renal disease with GFR < 30 mL/min or dialysis, Corticosteroid treatment > 10 mg/day prednisone equivalent for more than 2 weeks, systemic lupus or vasculitis with immunosuppressive therapy, hematopoietic stem cell allograft, or poorly controlled HIV or AIDS status. The comorbidities include at least one pathology among idiopathic pulmonary fibrosis, myopathy with a forced vital capacity, amyotrophic lateral sclerosis, Down syndrome, rare liver diseases, including autoimmune hepatitis, other rare diseases defined by the FSMR, obesity, diabetes, heart failure, COPD and chronic respiratory failure, complicated high blood pressure, and chronic renal failure.

## Data Availability

Raw data are held by the pharmaceutical companies. We are not authorized to provide publicly access to the data.
